# Anesthetic Considerations for the Transsphenoidal Resection of a Pituitary Tumor in an Acromegalic Patient: A Case Report

**DOI:** 10.7759/cureus.82447

**Published:** 2025-04-17

**Authors:** Kennedy P Kirkpatrick, Anvinh Nguyen

**Affiliations:** 1 Anesthesiology, Baylor College of Medicine, Houston, USA

**Keywords:** academic anesthesiology, acromegaly and diabetes, acromegaly and surgery, acromegaly comorbidities, anesthesia and neuro, neuro anesthesia, transsphenoidal neurosurgery

## Abstract

Acromegaly can result in many physiological changes that create multiple challenges for the anesthesiologist. Excess growth hormone secretion can result in altered facies, airway obstruction, ventilation challenges, and intubation difficulties. Moreover, hormonal changes can cause electrolyte imbalances, poor glycemic regulation, and hypertension. In addition, radial arterial invasive blood pressure monitoring can be relatively contraindicated given poor ulnar collateral circulation. In this case report, we discuss an acromegalic patient with severe facial, cardiovascular, and respiratory changes who successfully underwent transsphenoidal pituitary gland resection under general anesthesia. We describe our anesthetic approach toward caring for a patient with acromegaly.

## Introduction

Acromegaly is a condition caused by excess growth hormone secretion. Growth hormone over-secretion can result in thickened pharyngeal and laryngeal tissues, macroglossia, and enlarged facies [1]. Perioperatively, this can present significant challenges in developing an anesthetic plan. This can cause difficulties with bag-mask ventilation, intubation, invasive monitoring placement (arterial line), and create challenging extubation circumstances [2]. In fact, the incidence of difficult airways is significantly higher in acromegalic patients compared to normal patients [3]. Oftentimes, these patients require advanced airway techniques or tracheostomy to secure the airway [4-6]. This case report discusses a successful general anesthetic for the transsphenoidal resection of a pituitary tumor in an acromegalic patient.

## Case presentation

**Figure 1:**
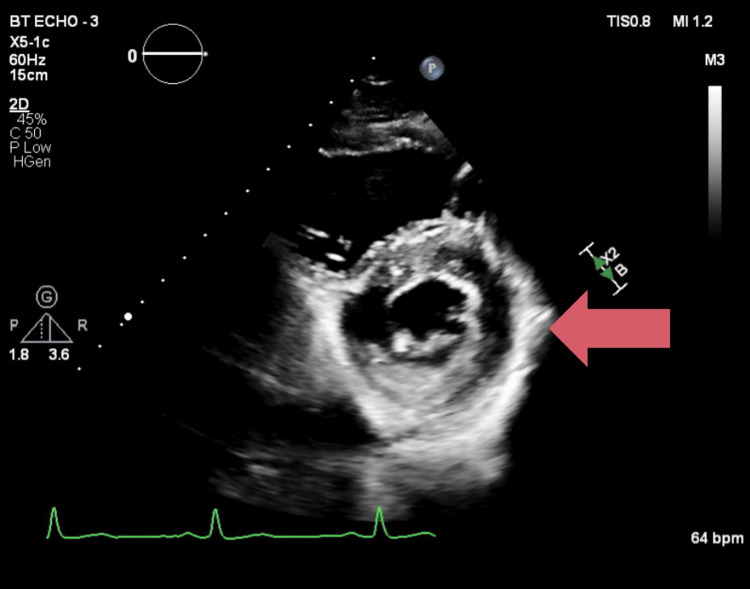
Transthoracic echocardiogram parasternal short axis view showing left ventricular concentric hypertrophy

A 52-year-old male presented for the transsphenoidal resection of a pituitary adenoma secreting excess growth hormone. Insulin-like growth factor 1 (IGF-1) was measured as 1067 ng/mL (reference range 74-255 ng/mL). Otherwise, his past medical history was significant for type II diabetes mellitus and hyperlipidemia. He took no medications at home and received none preoperatively. Physical examination of the patient was notable for enlarged hands and digits bilaterally, macroglossia, Mallampati 2 on airway exam, and prominent jaw structure with greater than 6 cm thyromental distance and adequate cervical spine range of motion in all directions. Preoperative labs were normal outside of mildly elevated glucose 145 mg/dL (reference range 70-110 mg/dL). Transthoracic echocardiography was significant for mild concentric remodeling and left ventricular ejection fraction of 60-64% with normal diastolic function (Figure 1).

**Figure 2:**
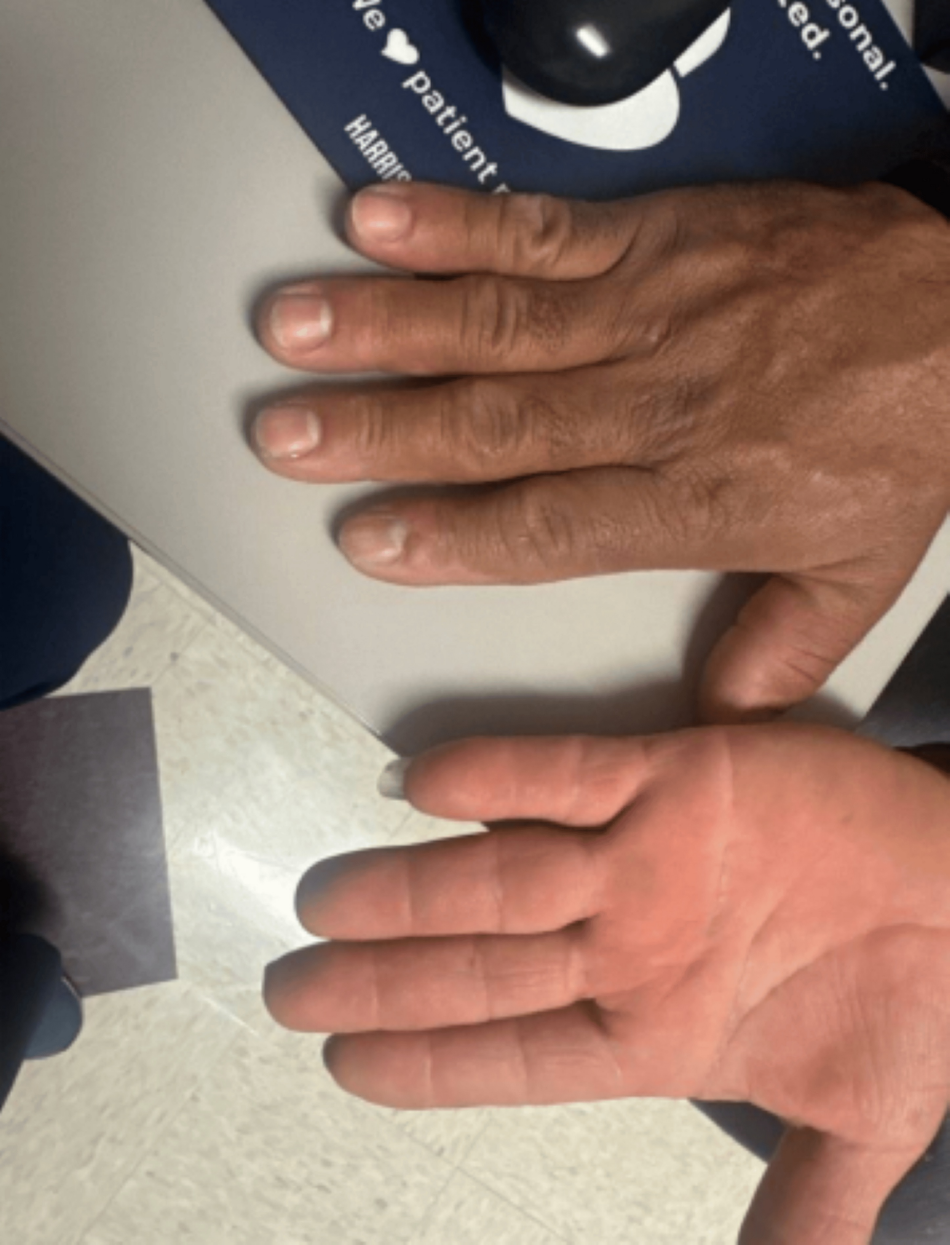
Acromegalic hands, hypertrophy of the digits bilaterally

Despite significant hypertrophy of the patient's hands (Figure 2), radial pulses were palpable bilaterally. A modified Allen's test with pulse oximetry was performed with adequate collateral flow in both upper extremities. Following this result, a preoperative arterial line was placed in the left radial artery.

Upon arrival to the operating room, no premedications were given. Vital signs were stable with pulse 66 bpm, blood pressure 141/71 mmHg, oxygen saturation (SPO2) 100% with preoxygenation occurring. Induction with 100 mcg fentanyl, 80 mg lidocaine, and 200 mg propofol was administered. After bag-mask ventilation was proven adequate with an oral airway, 60 mg rocuronium was given. Due to a reassuring airway exam, the decision was made to intubate using a C-Mac size 4 video laryngoscope (Karl Storz, Tuttlingen, Germany).

Significant macroglossia was noted while advancing a CMAC-4 blade. Hypertrophy of laryngeal tissues and global airway edema were visualized before reaching the epiglottis. A large, floppy epiglottis was visualized, and as the blade was inserted into the epiglottic vallecula, thickened vocal cords with a narrowed glottic opening were observed. The 8.0 mm endotracheal tube was passed through the vocal cords without trauma, and end-tidal CO2 was confirmed. Balanced maintenance of anesthesia was performed with 0.5 minimum alveolar concentration (MAC) sevoflurane in addition to a fentanyl (0.1 mcg/kg/min - 0.15 mcg/kg/min) and propofol (75 mcg/kg/hr - 125 mcg/kg/min) infusion. Infusions were turned off while the neurosurgical team was closing. Prior to extubation, an oropharyngeal airway (OPA) was placed, and return of spontaneous respiration with adequate minute ventilation was confirmed. As positive pressure ventilation is relatively contraindicated after the transsphenoidal approach, we optimized the patient position to improve pulmonary mechanics before proceeding. Extubation occurred without difficulty, and the patient was put on a non-rebreather mask for transport to the PACU with stable vital signs. The patient recovered in the PACU without any remarkable events.

## Discussion

Acromegalic patients can present many challenges from an anesthetic standpoint. A thorough airway exam is needed before proceeding to the operating room. The Mallampati score, tongue size, thyromental distance, neck mobility, and oral aperture can be useful in predicting a difficult airway; however, none of these tools is a perfect predictor of a difficult airway [2]. Our patient was easily maskable with an oral airway, but additional adjuncts (nasal trumpets, high-flow nasal cannula, etc.) may be necessary for airway patency. If an airway examination is not reassuring or the patient has a history of a difficult airway, options such as awake fiberoptic intubation or tracheostomy may be necessary [3-6].

In addition to being difficult to bag-mask ventilate, hypertrophy of the vocal cords and soft tissues in the airway itself often occurs, which can lead to challenges with intubation. Two retrospective studies cited difficult intubation in acromegalic patients at 10% and 30% [7,8]; however, the definition of "difficult intubation" in these studies was not well defined. A prospective study recently cited difficult intubation in acromegalic patients as two or more attempts, blade change, or the use of a gum-elastic bougie at 13% [3]. This study also noted grade 3 view intubation views with a Cormack-Lehane scale in 26% of patients, which was notably improved with backward, upward, rightward pressure (BURP) in 16% of these patients [3]. 

Predictors of difficult intubation in acromegalic patients have also been analyzed. In a study by Schmitt et al., Mallampati scores of 3 or 4 made up 71% of the study population; however, even among patients with a more reassuring Mallampati score, there was a negative predictive value of 20% for difficult intubation [3]. Sometimes awake fiberoptic intubation is necessary if there is significant concern for a difficult airway [7]. This is especially important in developing an anesthetic plan, as a reassuring Mallampati score can be misleading in acromegalic patients. Moreover, back-up difficult airway equipment should be readily available prior to the induction of anesthesia. Anesthesiologists should familiarize themselves with the conventional difficult airway algorithm as well [9].

Additionally, comorbidities related to long-standing exposure to increased growth hormone (GH) and IGF-1 levels, including hypertension (HTN) and abnormal glucose control, are prevalent in acromegaly. HTN in acromegalic patients is estimated at 35%, and severity directly correlates with GH levels [10]. The pathophysiology is not fully understood but is theorized to be related to the anti-natriuretic effects and sodium retention related to excess GH as well as insulin resistance and resultant sympathetic nervous system activity, decreased renal perfusion, and activation of the renin-angiotensin-aldosterone (RAAS) system [10]. GH and IGF receptors are also expressed in the kidneys and may directly stimulate sodium resorption at the distal tubules [11], which leads to another mechanism for hypervolemia and resultant HTN. In addition, to blunt the sympathetic activation from the resection of the pituitary mass, we had esmolol, nicardipine, and fentanyl ready in case tachycardia, hypertension, or significant pain occurred.

OSA occurs in 60-75% of acromegalic patients, which not only predisposes to difficult mask ventilation and intubation but also highlights another risk factor for the development of HTN [12]. Patients should be carefully risk-stratified, as HTN leading to cardiomyopathy and diastolic dysfunction is not uncommon in acromegalic patients. Cardiomyopathy in acromegalic patients is often seen in phases, with phase 1 being a hyperdynamic left ventricle with increased cardiac output, phase 2 showing progressive hypertrophy, and phase 3 showing both diastolic and systolic dysfunction leading to heart failure [12]. If EKG abnormalities are present preoperatively, with signs of left ventricular hypertrophy and left axis deviation, transthoracic echocardiography (TTE) should be considered if not already available. There are no current guidelines for cardiac testing among acromegalic patients, so it is at the discretion of the clinician to discern if additional tests are needed preoperatively based on risk stratification [13]. Intraoperatively, patient risk factors should be considered when choosing induction medications, the need for invasive monitoring, and controlling intubation, surgical stimulation, and extubation in order to prevent adverse cardiovascular effects.

Diabetes mellitus (DM) is another common comorbidity in acromegalic patients, which occurs at a higher rate than it does in patients without [11]. Treatment should be the same as in DM from other causes, and normalization of GH levels results in better glycemic control [14]. Intraoperative management should include careful monitoring of glucose levels through arterial blood gas analysis (ABG). Prior to surgical resection, hyperglycemia may be present, and insulin infusions may be needed for glycemic control. However, after resection, GH level normalization and the decrease in insulin resistance may make patients prone to hypoglycemia, especially if an insulin infusion was being used previously during the case. After pituitary mass resection, intra-operative glucose monitoring is essential to avoid hypoglycemia given the abrupt reduction in insulin levels. Postoperatively, handoffs to the PACU or ICU staff should include the importance of glucose monitoring and endocrinology consultation. 

Lastly, due to carpal tunnel hypertrophy, compression of the radial and ulnar arteries can occur, compromising collateral flow and limiting radial arterial line placement. Up to 50% of acromegalic patients have impaired ulnar artery circulation in one or both hands, leading to a risk of carpal tunnel syndrome [15]. Allen's test can be performed; however, it may be technically difficult and is less sensitive than Barbeau's test [16]. We carried out Barbeau's test, which is a modified Allen test performed with two pulse oximeters. One pulse oximeter is placed on the thumb of the patient's hand and another on the index finger. Then, compression of both the radial and ulnar arteries at the wrist is done, and once the hand appears blanched, pressure is released on one artery at a time, observing the pulse oximeter readings on each finger to see if the oxygen saturation quickly returns to normal, indicating adequate blood flow through the released artery. If oxygen saturation remains low after releasing one artery, it suggests inadequate collateral circulation through that pathway [16]. Our patient had adequate collateral flow bilaterally. However, alternative options, such as dorsalis pedis, can be investigated for continuous invasive blood pressure monitoring sites if collateral flow in the upper extremities does not exist. As always, the risks and benefits of invasive blood pressure monitoring should be weighed prior to cannulation.

## Conclusions

In summary, acromegaly can pose many challenges perioperatively. A thorough history and physical exam are needed prior to proceeding with surgery, although no specific airway exam has been shown to be a perfect predictor of a difficult airway in this patient population. In addition to airway challenges, invasive monitoring with arterial cannulation sites needs to be carefully analyzed preoperatively, as traditional radial arterial monitoring may not be feasible. 
